# Correlation between Low Temperature Adaptation and Oxidative Stress in *Saccharomyces cerevisiae*

**DOI:** 10.3389/fmicb.2016.01199

**Published:** 2016-08-03

**Authors:** Estéfani García-Ríos, Lucía Ramos-Alonso, José M. Guillamón

**Affiliations:** Food Biotechnology Department, Instituto de Agroquímica y Tecnología de Alimentos, Consejo Superior de Investigaciones CientíficasValencia, Spain

**Keywords:** thioredoxins, glutathione, correlation analysis, ROS accumulation, *MUP1*, *URM1*

## Abstract

Many factors, such as must composition, juice clarification, fermentation temperature, or inoculated yeast strain, strongly affect the alcoholic fermentation and aromatic profile of wine. As fermentation temperature is effectively controlled by the wine industry, low-temperature fermentation (10–15°C) is becoming more prevalent in order to produce white and “rosé” wines with more pronounced aromatic profiles. Elucidating the response to cold in *Saccharomyces cerevisiae* is of paramount importance for the selection or genetic improvement of wine strains. Previous research has shown the strong implication of oxidative stress response in adaptation to low temperature during the fermentation process. Here we aimed first to quantify the correlation between recovery after shock with different oxidants and cold, and then to detect the key genes involved in cold adaptation that belong to sulfur assimilation, peroxiredoxins, glutathione-glutaredoxins, and thioredoxins pathways. To do so, we analyzed the growth of knockouts from the EUROSCARF collection *S. cerevisiae* BY4743 strain at low and optimal temperatures. The growth rate of these knockouts, compared with the control, enabled us to identify the genes involved, which were also deleted and validated as key genes in the background of two commercial wine strains with a divergent phenotype in their low-temperature growth. We identified three genes, *AHP1, MUP1*, and *URM1*, whose deletion strongly impaired low-temperature growth.

## Introduction

Microorganisms constantly face environmental stimuli and stresses. The simplest response strategy to a stimulus is to monitor the environment and to respond directly to it using designated mechanisms. The environmental stress response in yeast is a complicated strategy in which responses to many stresses partially overlap (Gasch et al., 2000; Causton et al., 2001; Mitchell et al., 2009). Drops in ambient temperature are common in almost every ecological niche. In the yeast *Saccharomyces cerevisiae*, reductions in ambient temperature have widespread effects on growth and survival, which depend on the severity of stress. This is relevant for industrial yeast exploitations as several fermentations, like brewing and some wine fermentations, take place at around 12–15°C. In winemaking, fermentation at lower temperatures correlates with a fresh character and fruity notes in wines, and reduces the risk of bacterial contamination and the production of volatile acids (Beltran et al., 2002; Torija et al., 2003; Molina et al., 2007). The use of low temperature during the fermentation process improves product quality, but also prolongs the time needed to complete fermentation and, therefore, increases the economic cost and energy requirements. Industry is clearly interested in developing yeast strains with an enhanced capability to ferment at low temperatures. It is well-known that cold induces biochemical, biophysical and physiological changes to cells. Cold strengthens the interactions between the two strands of DNA and the secondary structure of mRNA, so transcription and translation are impaired (Jones and Inouye, 1996). The fluidity of the lipid bilayer of membranes also diminishes and their rigidity increases, which decreases transport through cell membranes and increases passive permeability (Redón et al., 2011). Protein folding speed slows down as conformational instability increases. Cold can also induce protein denaturation and enzymatic activity generally decreases (Schade et al., 2004; Murata et al., 2006; Aguilera et al., 2007).

The release of reactive oxygen species (ROS) and the generation of oxidative stress can negatively impact yeast cell survival. ROS can be produced in the course of normal aerobic metabolism or when an organism is exposed to a variety of stress conditions. These include hydrogen peroxide (H_2_O_2_), the hydroxyl radical (OH^•^), and the superoxide anion (O2^•−^), which can damage proteins, lipids, carbohydrates, and DNA (Herrero et al., 2008), which can lead to cell death. However, cells possess a variety of defenses, including cell-cycle delay (Flattery-O'Brien et al., 1993), the induction of enzymes, such as catalases, peroxidases, and superoxide dismutases, and the synthesis of antioxidants like glutathione, vitamins C and E, and ubiquinol (Jamieson, 1998; Morano et al., 2012). In the last few years, some studies have connected low temperature with oxidative stress. Zhang et al. (2003) showed that a rapid downshift in the growth temperature of *S. cerevisiae* from 30 to 10°C increased intracellular hydrogen peroxide (H_2_O_2_) levels and raised the transcript levels of antioxidant genes *SOD1, CTT1*, and *GSH1*. Schade et al. (2004) also reported that a drop in the temperature induced a set of genes involved in oxidative stress response and implicated in detoxification processes, including *GTT2* (glutathione transferase), *HYR1* and *GPX1* (glutathione peroxidase isoforms), *TTR1* (glutaredoxin), and *PRX1* (thioredoxin peroxidase). Likewise, by means of an integrative analysis of transcriptome with metabolome and proteome data, recent studies by our group have revealed the up-regulation of the sulfur assimilation pathway and glutathione biosynthesis during adaptation to cold (García-Ríos et al., 2014). The sulfur pathway incorporates extracellular sulfate into several key sulfur-containing compounds, including methionine, cysteine, homocysteine and S-adenosylmethionine (SAM; Hickman et al., 2011). Sulfur assimilation induction is easily understood in the oxidative stress context as cysteine is a component of molecules like glutathione, glutaredoxin and thioredoxin, which were all induced in response to oxidative stress (Sha et al., 2013), while methionine acts as a ROS scavenger (Campbell et al., 2016).

After considering all these data, this work aimed to identify the correlation between low-temperature growth and recovery after oxidative stress shock, and also the detection of the key genes related to the oxidative stress response, which play an important role in the adaptation of *S. cerevisiae* to low temperature. To achieve this objective, we analyzed the growth of several knockouts of sulfur assimilation, peroxiredoxins, glutathione-glutaredoxins and thioredoxins pathways in the lab strain BY 4743 at 15°C and 28°C. The first screening of this laboratory strain enabled us to select genes in order to delete them from the genetic background of two commercial wine strains with divergent phenotypes in both low temperature and oxidative stress responses.

## Methods

### Yeast strains and media

In this study, 40 *S. cerevisiae* strains were used, which were mainly industrial. These strains were typed by their interdelta sequences (Legras and Karst, 2003), and thus named according to their delta pattern (from P1 to P40). All the strains used in the correlation study herein are detailed in Table 1. Inocula were prepared by introducing one single colony from the pure cultures of each strain into 5 ml of YPD medium (1% yeast extract, 2% peptone and 2% glucose). After 24 h of incubation at 30°C, the volume required to obtain a concentration of about 2 × 10^6^ cells/ml was inoculated in the different media. Correct inoculation size was always confirmed by surface spread on YPD agar plates. These yeast suspensions were used to inoculate the different experiments as described below. Forty mutants (Supplementary Table 1) that belonged to the sulfur assimilation pathway and oxidative stress response of laboratory strain BY4743 (MATa/α, his3Δ 1; leu2Δ 0; lys2Δ 0; ura3Δ), from the EUROSCARF collection (Frankfurt, Germany), were also used.

**Table 1:** **The ***Saccharomyces cerevisiae*** strains used in this study**.

**Code name**	**Strain designation**	**Origin/Source**
P1	Lalvin®ICVD254	Lallemand Inc. (France)
P2	Uvaferm®WAM	Lallemand Inc. (France)
P3	Lalvin®ICVD80	Lallemand Inc. (France)
P4	Lalvin®Rhone2056	Lallemand Inc. (France)
P5	Lalvin®ICVGRE	Lallemand Inc. (France)
P6	Lalvin®EC1118	Lallemand Inc. (France)
P7	Lalvin®ICVD47	Lallemand Inc. (France)
P8	Uvaferm®CEG	Lallemand Inc. (France)
P9	Lalvin®Rhone2323	Lallemand Inc. (France)
P10	Uvaferm®BC	Lallemand Inc. (France)
P11	Uvaferm®VRB	Lallemand Inc. (France)
P12	Uvaferm®43	Lallemand Inc. (France)
P13	CrossEvolution®	Lallemand Inc. (France)
P14	Lalvin®71B	Lallemand Inc. (France)
P15	Lalvin®BM45	Lallemand Inc. (France)
P16	Enoferm®M1	Lallemand Inc. (France)
P17	Enoferm®M2	Lallemand Inc. (France)
P18	Uvaferm®BDX	Lallemand Inc. (France)
P19	Uvaferm®CM	Lallemand Inc. (France)
P20	Lalvin®ICVD21	Lallemand Inc. (France)
P21	Lalvin®Rhone2226	Lallemand Inc. (France)
P22	Lalvin®CY3079	Lallemand Inc. (France)
P23	^*^	Lallemand Inc. (France)
P24	^*^	Lallemand Inc. (France)
P25	^*^	Lallemand Inc. (France)
P26	^*^	Lallemand Inc. (France)
P27	^*^	Lallemand Inc. (France)
P28	QA23	Lallemand Inc. (France)
P29	S288c	Lab Strain
P30	AJ4	Lallemand Inc. (France)
P31	RVA	Agrovin company (Ciudad Real, Spain)
P32	T73	Wine (Alicante, Spain)
P33	BMV60	Wine (Murviedro Wineries, Valencia, Spain)
P34	PE35M	Masato (Greater San Marcos University, Lima, Peru)
P35	Temohaya-26	Agave juice (Technological Institute of Durango, Mexico)
P36	CPE7	Cachaça (Federal University of Minas Gerais, Brazil)
P37	Kyokai no.7	Sake (Japan)
P38	GB-FlorC	Jerez wine (González-Byass Wineries, Jerez, Spain)
P39	CECT10131	*Centaurea alba* (CECT, Spain)
P40	Fermol Grand Rouge Nature	AEB group (Cape Town, South Africa)

* *These strains have no commercial name as they are already in the development stage*.

The growth media selected for the experiments were YPD (glucose 20 g L^−1^, peptone 20 g L^−1^, yeast extract 10 g L^−1^) and synthetic grape must (SM). The latter was derived from that described by Quirós et al. (2013). The SM composition included 200 g L^−1^ of sugars (100 g L^−1^ glucose + 100 g L^−1^ fructose), 6 g L^−1^ of malic acid, 6 g L^−1^ of citric acid, 1.7 g L^−1^ of yeast nitrogen base (YNB) without ammonium and amino acids, anaerobic factors (0.015 g L^−1^ ergosterol, 0.005 g L^−1^ sodium oleate, and 0.5 mL L^−1^ tween 80) and 0.060 g L^−1^ of potassium disulfite. The assimilable nitrogen source used was 0.3 g N L^−1^ (0.12 g N L^−1^ as ammonium chloride and 0.18 g N L^−1^ in an amino acid form; the proportion of each amino acid was administered as previously proposed by Riou et al., 1997). The sporulation medium was KAc (potassium acetate 1%, agar 2%).

To test differential stress oxidative resistance, yeast cells were incubated in PBS with 4 mM of peroxide of hydrogen, 2 mM of menadione, 0.5 mM of cumene hydroperoxide, or 0.5 mM of tert-butyl hydroperoxide for 1 h at 28°C. These concentrations were selected after testing different amount of oxidants. The selected concentration produced a clear growth impairment but did not jeopardize the viability of the culture, such as was described in García-Ríos et al. (2014) for the peroxide of hydrogen. After this oxidative shock, cells were centrifuged at 10,000 rpm for 3 min at room temperature and inoculated in SM at 28°C.

### Growth conditions

Growth was monitored by determining optical density at 600 nm in a SPECTROstar Omega instrument (BMG Labtech, Offenburg, Germany). Measurements were taken every 30 min for 4 days after 20-s pre-shaking for all the experiments. At low temperatures (12–15°C), however, microplates had to be incubated outside the spectrophotometer to then be placed inside before being measured (every 3 h for 14 days). Microplate wells were filled with the required volume of inoculum and 0.25 ml of YPD or SM medium to always ensure an initial OD of approximately 0.1 (inoculum level of about 10^6^ cells mL^−1^). For each experimental series, non-inoculated wells were also included in the microplate to determine, and to therefore subtract, the noise signal. All the experiments were carried out in triplicate. Growth parameters were calculated from each treatment by directly fitting OD measurements vs. time to the reparametrized Gompertz equation proposed by Zwietering et al. (1990):

$$\text{y}={\text{D}}^{*}\text{exp }\{-\text{exp}[((\mu_{\text{max}}*\text{e})/\text{D})*(\lambda -\text{t}))+1]\}$$

where y = ln(OD_t_/OD_0_), OD_0_ is the initial OD and OD_t_ is the OD at time t; D = ln(OD_t_/OD_0_) is the asymptotic maximum, μ_max_ is the maximum specific growth rate (h^−1^), and λ is the lag phase period (h) (Aguilera et al., 2007). Generation time (GT) was calculated using the equation GT = ln2/μ_max_. The overall yeast growth was estimated as the area under the OD vs. time curve (70 and 250 h at 28 and 15°C, respectively). This parameter was calculated by integration using the OriginPro 8.0 software (OriginLab Corp., Northampton, MA).

### Construction of mutants in the background of a wine strain

We constructed P5 and P24 homozygous and homothallic strains by autodiploidization of one ascospore and tested the fitness of the monosporic culture by comparing with the parental strain. Heterozygous mutants were constructed using the short flanking homology (SFH) method (Güldener et al., 1996) by transforming both wine strains according to the lithium acetate procedure (Daniel Gietz and Woods, 2002) with a PCR fragment obtained by amplifying the KanMX4 cassette and flanking regions (about 500-pb upstream and downstream) from the corresponding mutant strain in the BY4743 background. After transformation, strains were selected using Geneticin (G418), added to the YPD solid media at a concentration of 200 mg L^−1^. The total DNA from the transformants resistant to G418 was analyzed by PCR using the primers upstream and downstream of the deleted region combined with the primers inside KanMX.

The homozygous mutants were constructed by sporulating in potassium acetate medium (KAc) the heterozygous mutants and testing spores for G418 resistance. As expected, the geneticin resistance feature segregated 2:2. Since the original strain was homothallic, the strains recovered from the segregation analysis plates were spontaneous autodiploids, and were homozygous for the corresponding gene deletion, as verified by PCR.

### Synthetic wine must fermentation

Fermentations were performed at 28 and 15°C with continuous orbital shaking at 100 rpm. Fermentations were carried out in laboratory-scale fermenters using 100 mL bottles filled with 60 mL of SM. Fermentations were monitored by media density (g L^−1^) in a densitometer (Densito 30PX, Mettler Toledo, Switzerland). Fermentations were considered complete when density reached 995 g L^−1^. Yeast cell growth was determined by absorbance at 600 nm and by plating on YPD. The kinetics of these fermentations was estimated by calculating the time needed to ferment 100% (T100) of sugars in SM.

### Statistical analysis

All the experiments were carried out at least in triplicate. Physiological data and correlation tests were analyzed by the Sigma Plot 12.5 software and the results are expressed as mean and standard deviation. To evaluate statistical significance, tailed t-student tests were applied with a *p*-value of 0.05. Phenotypic data were fitted to the reparametrized Gompertz model by non-linear least-squares fitting using the Gauss-Newton algorithm as implemented in the nls function in the R statistical software, v.3.0.

## Results

### Correlation analysis between low temperature and oxidative stress

**Figure 1:**
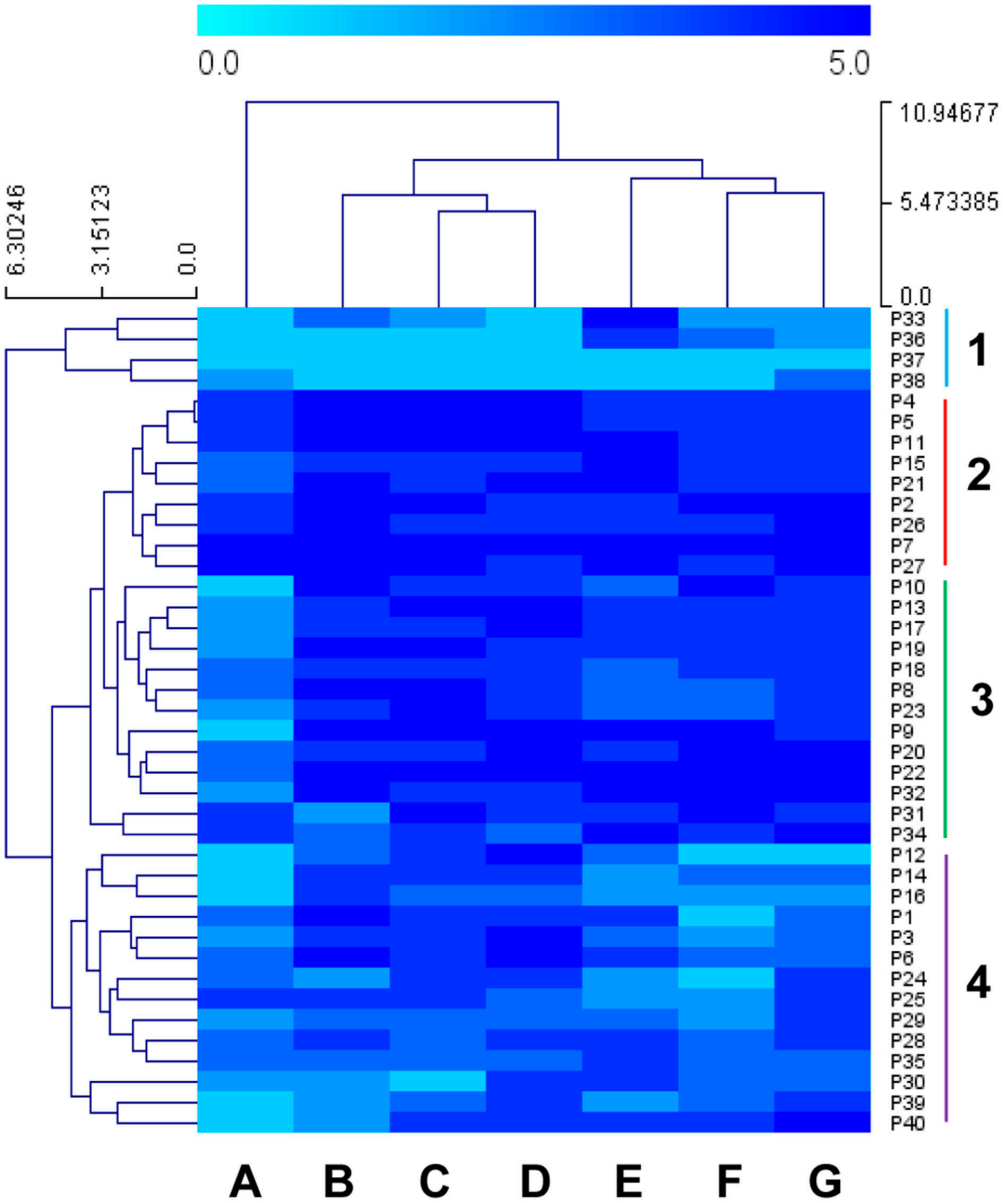
**Heatmap illustrating the area under the curve (AUC) of 40 strains under seven different conditions**. Growth experiments were performed in synthetic must (SM) at 12 and 15°C for the low temperature analysis and SM at 28°C after oxidative shock to test the recovery capability. In order to normalize the different conditions, strains were divided into five groups depending on the minimum and maximum AUC value of the population per experiment. (A) Cumene hydroperoxide 0.5 mM at 28°C, (B) SM at 15°C, (C) SM at 12°C, (D) Hydrogen peroxide 4 mM at 28°C, (E) SM at 28°C, (F) Menadione 2 mM at 28°C, (G) Tert butyl hydroperoxide at 28°C. Sensitivity is indicated in light blue, and resistance in dark blue. The four groups separated by the Hierarchical clustering analysis (HCL) are marked by colors.

In order to assess the correlation between low temperature and oxidative stress, hierarchical clustering analyses were performed using Euclidean distances with the area under the OD vs. time curve (AUC) of each growth experiment (using the mean values of biological replicates). Figure 1 shows the clustering of the 40 strains under the seven assayed conditions. Low-temperature (12 and 15°C) growths clustered together with recovery after H_2_O_2_ shock. A second cluster was observed between growth after both shocks with oxidants menadione and tert butyl hydroperoxide and growth in SM at 28°C, which highlights the weak correlation between the recovery of these two oxidant shocks and the low temperature during fermentations. Finally, growth after cumene hydroperoxide shock clustered alone. By considering sample clustering, we saw four major groups. Group 1 consisted in four strains isolated from two wineries, sugar cane and sake fermentation, and presented the worst recoveries after oxidative stress. Groups 2 and 3 were integrated mainly by wine yeasts and one strain isolated from the masato fermentation (P34). This set was characterized by good recoveries after oxidative shock, especially group 2. The characteristic by which these groups mainly differed was the behavior noted against cumene hydroperoxide since Group 2 presented better recoveries than Group 3. Group 4 was a mixture of strains from different environments (industrial, lab, agave, blueberries and *Centaurea alba* flowers) that displayed mosaic behavior to the different assayed conditions.

**Figure 2:**
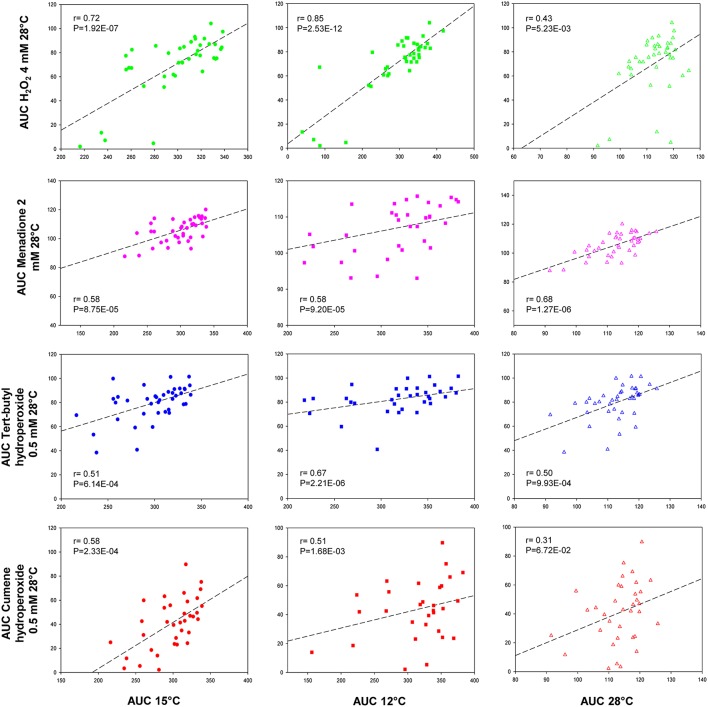
**Correlation analysis between temperature and oxidant agents**. The mean AUC value in SM at 15, 12, and 28°C was plotted against the mean AUC value under each oxidative condition. Linear regression (black line) is displayed. The squared Pearson correlation coefficient (r) and the *p*-value are provided in this figure.

To quantitatively measure the correlations between growth temperatures (12, 15, and 28°C) and the capacity to deal with oxidative stress, we performed several Pearson correlation tests (Figure 2). It is noteworthy that all the correlations were positive, irrespectively of the oxidant agent used. However, the strongest correlations were found between H_2_O_2_/12°C (*r* = 0.85) and H_2_O_2_/15°C (*r* = 0.72), and this *r*-value lowered (*r* = 0.43) when the correlation was done with the control temperature (28°C/H_2_O_2_). Although significant in many cases, the correlations between low temperature and the rest of the oxidant agents were moderate and the differences between temperatures were not as clear as in H_2_O_2_.

### Screening of genes related with the oxidative stress response and the sulfur assimilation pathway in the BY4743 mutant collection

**Figure 3:**
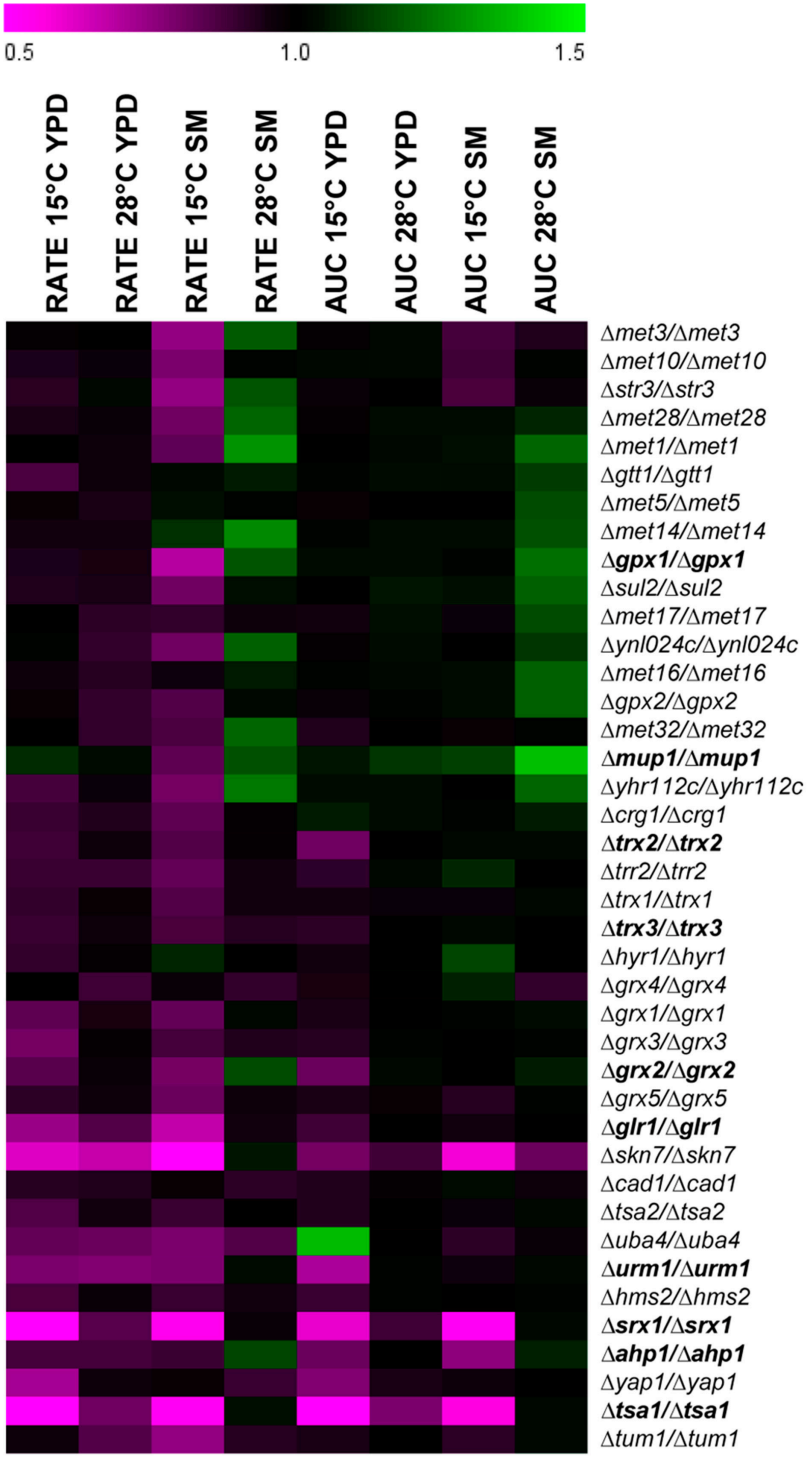
**Growth parameters (rate and AUC) of 40 mutants strains of the BY4743 collection related to oxidative stress response**. The values are relative compared with control strain BY4743. Values below 1 meant that the parameter was affected compared with the control strain. Sensitivity is indicated in purple, and resistance in green. The mutants in bold were selected for their construction in the wine strains.

Forty mutants of the BY4743 collection were chosen to phenotype them regarding their growth capacity at 15 and 28°C in YPD and SM (Figure 3). These genes belonged to four biochemical pathways: sulfur assimilation, peroxiredoxins, glutathione-glutaredoxins, and thioredoxins. Figure 3 shows the relative AUC and the maximum specific growth rates (μ_max_) compared with the BY4743 strain for each condition. Values below 1 meant that the mutant strain had impaired growth compared with the control strain. To select a gene as being determinant for low-temperature growth, its mutant strain had to show significantly impaired growth in at least two of the four growth conditions at 15°C (rate or AUC in YPD or SM) and for any of the growth conditions at 28°C. According to this criterion, 10 genes (Table 2) were selected: *TRX3, AHP1, TSA1, SRX1, GLR1, GRX2, TRX2, GPX1, URM1*, and *MUP1*. Fortunately, the four representative pathways related with oxidative stress, which we aimed to study, had several candidates among the 10 chosen genes.

**Table 2:** **Selected genes with an affected phenotype at low temperature in the BY4743 strain**.

**Gene name**	**Description**	**Function**
*MUP1/*YGR055W	High-affinity methionine permease	Methionine and cysteine uptake
*GPX1/*YKL026C	Glutathione peroxidase	Protects cells from phospholipid and non-phospholipid hydroperoxides during oxidative stress
*TRX2/*YGR209C	Cytoplasmic thioredoxin isoenzyme	Protects cells against oxidative and reductive stress
*TRX3/*YCR083W	Mitochondrial thioredoxin	Mantains the redox homeostasis of the cell
*GRX1/*YCL035C	Glutathione-dependent disulfide oxidoreductase	Hydroperoxide and superoxide-radical responsive, protects cells from oxidative damage
*GLR1/*YPL091W	Glutathione reductase	Converts oxidized glutathione to reduced glutathione
*URM1/*YIL008W	Ubiquitin-related modifier	Receives sulfur from E1-like enzyme Uba4p and transfers it to tRNA
*AHP1/*YLR109W	Alkyl hydroperoxide reductase	Reduces hydroperoxides to protect against oxidative damage
*TSA1/*YML028W	Thioredoxin peroxidase	Acts as both ribosome-associated and free cytoplasmic antioxidant
*SRX1/*YKL086W	Sulfiredoxin	Contributes to oxidative stress resistance by reducing the cysteine-sulfinic acid groups in peroxiredoxin Tsa1p

### Construction of oxidative stress response mutants in two industrial *S. cerevisiae* strains with a divergent phenotype at low temperature

In a previous work (García-Ríos et al., 2014), a collection of industrial wine strains was phenotyped according to their capacity to grow and ferment at low temperature, and two strains (P5 and P24) were selected that displayed divergent behavior. The P5 strain showed a much better fitness than P24 when grown at a low temperature, while both strains exhibited average growth at 28°C. These strains also exhibited the different capacity to deal with oxidative stress, which once again correlated well with its growth capacity at low temperature (P5 also obtained better growth recovery after oxidative stress). These previous data were confirmed in this study with the results shown in Figure 1: P5 clustered with Group 2, the more resistant strains for all the assayed conditions, while P24 belonged to Cluster 4, which is integrated by strains with worse recoveries against oxidative stress.

**Figure 4:**
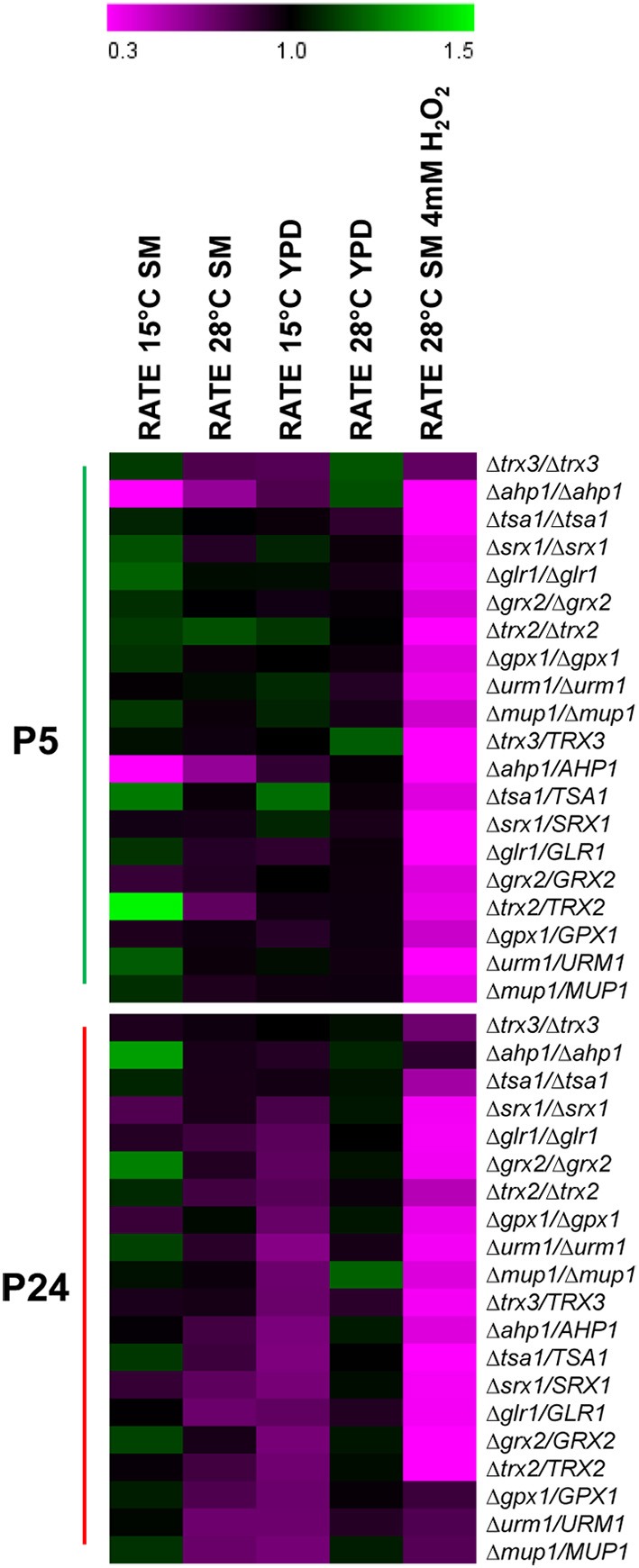
**Relative growth rate of the mutants constructed in the background of a wine strain**. The selected genes of the BY4743 collection were deleted in strains P5 and P24 and their fitness was tested under different conditions. Values below 1 meant that the parameter was affected compared with the control strain. Sensitivity is indicated in purple, and resistance in green.

The 10 genes that provoked a growth defect at low temperature in the BY4743 background were selected to construct heterozyogous and homozygous mutant strains in both wine strains. Heterozygous mutants were constructed by deleting one of the two copies in the parental strains (diploid). After the sporulation of heterozygous strains, homozygous mutants were obtained by autodiploidization of one spore, which harbored the deleted allele (G418 resistant). We analyzed the growth capacity of the 20 homo- and hetero-zygous mutants of each wine strain at 15 and 28°C in YPD and SM media. We also tested the recovery fitness of the mutants after the oxidative stress caused by H_2_O_2_. Figure 4 shows a heat-map of the relative growth rate (μ_max_) of the mutants compared with each parental strain. Values below 1 meant that the mutant strain had impaired growth compared with the parental strain. It is noteworthy, but not surprising, that all the homozygous and heterozygous mutants constructed in both strains showed significant growth impairment after oxidative shock. Moreover, practically all these mutants constructed in the background of P24 also displayed impaired growth in YPD at 15°C, whereas the growth of very few mutants of P5 was significantly affected under this condition. Curiously enough, the most affected mutants in P5 (*trx3* and *ahp1*) were the less affected mutants in P24. This result reinforces the correlation between low temperature and oxidative stress in the P24 strain because the deletion of genes involved in response to this latter stress also affected its growth capacity at low temperature. However, this correlation was not confirmed in the P5 strain, in which most mutants were not affected when grown at a low temperature. The only gene whose deletion in both heterozygosity and homozygosity provoked growth impairment in the P5 strain was *AHP1*, which encodes a thiol-specific peroxiredoxin that reduces hydroperoxides to protect against oxidative damage (Trivelli et al., 2003).

It was noteworthy that mutants' growth capacity was not so widely affected in SM as in YPD at low temperature. This is striking because SM is a much more stressful medium because of high sugar and low nitrogen concentrations, low pH, etc. Together with growth capacity, we were also interested in these mutants' fermentation capacity because they were constructed in two strains used industrially for wine fermentations.

### Fermentation kinetics of oxidative stress response mutants

**Figure 5:**
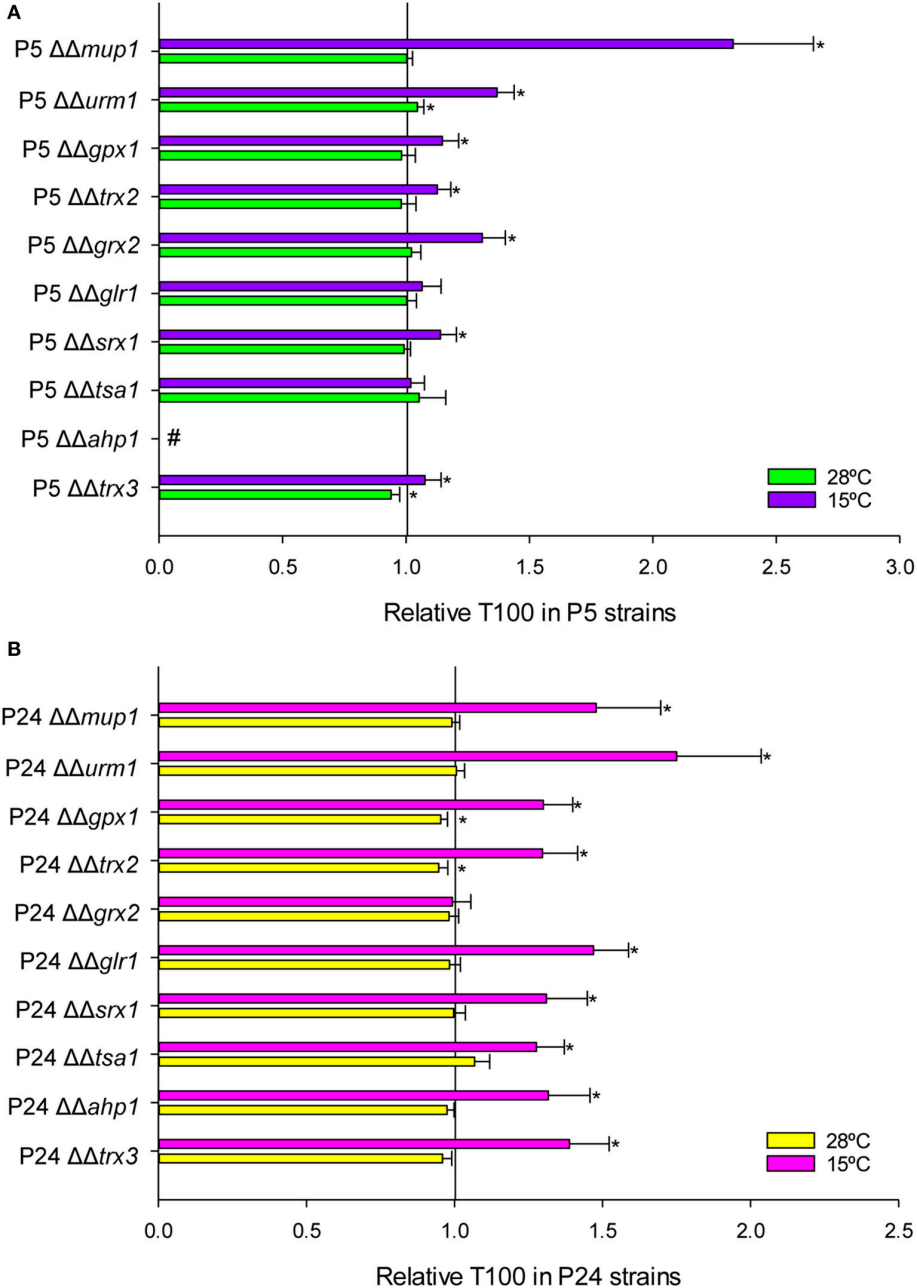
**Fermentation kinetics in the mutant strains constructed in P5 (A) and P24 (B) at 15 and 28°C**. T100 is the time needed to consume the total amount of sugars present in the must. The T100 value was compared with the control normalized as value 1. ^*^Significant differences (*p* ≤ 0.05) compared with the control at the same temperature. ^#^Indicates a stuck fermentation before T100.

Fermentation activity was estimated by calculating the time required to ferment 100% (T100) of sugars in the SM at 15 and 28°C. T100 values below 1 indicated faster sugar consumption, while T100 values over 1 implied a delayed fermentation end compared with control strains P5 and P24. In this case, only the homozygous mutants were analyzed because we expected a higher impact on fermentation activity as a result of the deletion of both copies of a gene. Figure 5A shows the relative T100 of the P5 deletant strains. Conversely to growth data, a delay in the fermentation process at low temperature took place with most strains, except for genes *TSA1* and *GLR1*. The mutants with the most affected phenotypes were *MUP1*, and specially *AHP1*, whose deletion rendered this mutant incapable of ending fermentation at both temperatures. All the mutants constructed in the P24 background (Figure 5B), except *GRX2*, presented an affected phenotype at low temperature, but were not affected or barely impaired at 28°C. Generally speaking, end of fermentation was delayed longer for the mutants constructed in P24 than for the P5 mutants. The gene with the strongest impact was *URM1*, followed by *MUP1*. Conversely to that observed in the P5 strain, deletion of *AHP1* in P24 impaired fermentation activity at low temperature, but was unaffected at 28°C.

## Discussion

In a previous study we selected two wine strains (P5 and P24) based on their divergent phenotype at a low, but not at optimum, temperature. We followed a global approach by using comparative transcriptomics, proteomics, and genome sequencing between both strains for the purpose of clearing up the molecular basis under this divergent phenotype (García-Ríos et al., 2014). The transcriptomics analysis revealed key changes in the sulfur assimilation pathway and in other genes involved in oxidative stress defense at low temperature. So we hypothesized that low temperature adaptation and oxidative stress can share common protective mechanisms.

Hence the present work aimed to investigate the relationship between low-temperature adaptation and recovery after oxidative stress shock. For this purpose, we analyzed the growth of 40 *S. cerevisiae* strains under several temperature and oxidative stress conditions to establish a mathematical correlation between them. Our results revealed that low-temperature growth correlated highly with the behavior of cells against most of the assayed oxidants, but particularly with hydrogen peroxide. Menadione obtained the worst correlation as the Pearson value was higher compared with 28°C than in the cold. Flattery-O'Brien et al. (1993) already reported a different response in *S. cerevisiae* cells treated with menadione in comparison to the same cells treated with hydrogen peroxide because these agents are thought to generate different ROS.

Thus our results clearly correlated low temperature and the oxidative stress produced by some strong oxidant molecules. However, one question remains: why does low temperature exert a stronger oxidative situation in cells than an optimum temperature? Previous studies have demonstrated that a downward shift in the growth temperature of *S. cerevisiae* from 30 to 10°C increases intracellular H_2_O_2_ levels (Zhang et al., 2003) and induces an antioxidant response (Schade et al., 2004). Recently, two independent studies (Paget et al., 2014; Ballester-Tomás et al., 2015) proved that low temperature produced a redox imbalance that needs to be corrected by the dynamic regulation of the NAD(P)/NAD(P)H ratio and the intracellular levels of these co-enzymes. Paget et al. (2014) corrected this redox imbalance by increasing glycerol accumulation or cytosolic acetaldehyde production by deleting *GUT2* and *ADH3*, respectively. Ballester-Tomás et al. (2015) compensated this redox imbalance by overexpressing the *GDH2*-encoded glutamate dehydrogenase gene, which increased NADH oxidation. In both strategies, the *S. cerevisiae* strains displayed better fitness at low temperature, and both studies identified redox co-enzymes as key factors that governed yeast cold growth.

In order to assess a direct implication of the cellular mechanisms involved in the oxidative stress response during adaptation at low temperature, different homozygous mutants of the BY4743 strain were tested for their fitness to grow at low temperature. Although most of these genes showed impaired growth for any of the conditions tested at low temperature, we only selected 10 genes, those that showed a severe growth defect, to construct heterozygous and homozygous mutants in both wine strains with different competitiveness at low temperature. One striking result was that the growth of practically all the heterozygous mutants (deletion of one copy) was strongly affected after oxidative shock, and exhibited a haploinsufficient growth defect. Haploinsufficiency is defined as a dominant phenotype in diploid organisms that are heterozygous for a loss-of-function allele. Deutschbauer et al. (2005) used the complete set of *S. cerevisiae* heterozygous deletion strains to survey the genome for haploinsufficiency by fitness profiling in rich (YPD) and minimal media. This assay revealed that approximately only 3% of all the 5900 tested genes were haploinsufficient for growth in YPD, and concluded that haploinsufficiency is remarkably rare in *S. cerevisiae*. However, in this set of genes related with oxidative stress, the retention of a single copy, which implies reduced gene dosage, was not enough to fight against and recover after an oxidative stress response. This denotes the importance of proper protein production on these oxidative response pathways. Likewise, practically all the heterozygous mutants of P24 also showed haploinsufficiency when grown at 15°C in YPD, which revealed the dependence of this strain on a suitable oxidative stress protection system for growth at low temperature. Conversely, few heterozygous or homozygous mutants of P5 displayed impaired growth at low temperature, which correlates with its better fitness for this condition and denotes a lower dependency of the oxidative stress protection. It is also interesting the different response obtained in both media, wherein growth capacity was less affected in SM. Even some homozygous and heterozygous mutants showed significant growth improvements (higher μ_max_) when were grown in SM at 15°C. *S. cerevisiae* is an organism highly regulated with many genes and pathways to sense and respond appropriately to changing environmental conditions. The deletion of some genes that relaxed these strict controls might be beneficial in a stressful and complex medium as SM.

As P5 and P24 have a wine origin, we also tested the fermentation capacity of the homozygous mutants constructed in these strains. Similarly to growth rate, fermentation ended with a significant delay for most P24 mutants at 15°C, but not at 28°C. Regarding the P5 mutants, most of the genes also impacted fermentation activity at low temperature, but deletion of *MUP1* was quite remarkable as it increased the fermentation time by more than 2.5-fold. *MUP1* is a high-affinity methionine permease that is also involved in cysteine uptake (Kosugi et al., 2001). As explained above, this strain showed a very active up-regulated sulfur assimilation pathway at low temperature, and the phenotype observed for *mup1* also evidenced the need for sulfur amino acid uptake during wine fermentation at low temperature. Another gene to consider was *AHP1*, a thiol-specific peroxiredoxin that reduces hydroperoxides (Trivelli et al., 2003). The deletion of this gene in P5 strongly impaired the growth rate and produced a stuck fermentation in the strain P5 at both temperatures. However, this phenotype was temperature-independent and strain-dependent because the deletion of *AHP1* in P24 did not lead to the greatest reduction in growth and fermentation fitness. Strangely enough, the deletion with the strongest impact on the fermentation activity of P24 was *URM1*, a gene which encodes an ubiquitin-related protein that serves as a post-translational modification of other proteins. The Urm1p conjugation has been implicated in the budding process and in nutrient sensing. Goehring et al. (2003) suggested that the conjugation of Urm1p to Ahp1p could regulate the Ahp1p function in the antioxidant stress response in *S. cerevisiae*. Nevertheless, if the Ahp1p-Urm1p conjugate was necessary for a proper antioxidant response, why did the disruption of one of these genes strongly affect the fitness in P5, but not in P24, and vice versa? Further future studies will be necessary to answer this question.

In conclusion, we clearly established herein a strong correlation between low temperature fitness and oxidative stress resistance in *S. cerevisiae*. Our hypothesis is that growing this yeast at a suboptimal temperature raises the intracellular levels of ROS and induces an antioxidant response. The fitter strains to fight against this oxidative stress are also the strains that display better growth and fermentation performance at low temperature. This correlation is also very interesting from an applied point of view because it could be a trait for future selections of industrial cryotolerant strains or for the genetic improvement of them. We have recently obtained an improved wine strain to ferment at low temperature by growing during many generations under low-temperature selective pressure (López-Malo et al., 2015). An alternative strategy could be the long-term culture of these strains in the presence of oxidants to obtain fitter genetic variants to cope with the oxidative stress and to better adapt to low temperature. In order to assess this correlation at the molecular level, we constructed mutants of the genes involved on the main antioxidant response pathways in two wine strains with a divergent phenotype at low temperature. The growth and fermentation fitness of P24 was seen to strongly depend on an optimal oxidative stress response. With P5, this being the strain that displayed better competitiveness at low temperature, the deletion of key oxidative stress defense genes did not lead to a general reduction in its fitness. As low temperature adaptation is a trait that is regulated by many complex mechanisms at different levels in the cell, P5 must cope with this stress by other alternative mechanisms of stress response. However, the phenotype analysis of the mutants in this strain revealed the importance of the permease of sulfur amino acids *MUP1* to grow and ferment at low temperature and the paramount role of peroxiredoxin *AHP1* in the fermentation process.

## Author contributions

EG conducted the experiments, analyzed the data, and wrote the manuscript. LR performed the experiments. JG conceived the study, participated in the study design, and wrote the manuscript. All the authors read and approved the final manuscript.

## Funding

This work has been financially supported from the Spanish Government through MINECO and FEDER funds (AGL2013-47300-C3-3-R and PCIN-2015-143 grants) and from Generalitat Valenciana through PROMETEOII/2014/042 grant, awarded to JG. This study has been carried out in the context of the European Project ERA-IB “YeastTempTation” EG also thanks the Spanish government for an FPI grant BES-2011-044498.

### Conflict of interest statement

The authors declare that the research was conducted in the absence of any commercial or financial relationships that could be construed as a potential conflict of interest.
